# Governing digital transformation in ports: Policy learning from the 5G-LOGINNOV project

**DOI:** 10.12688/openreseurope.21641.3

**Published:** 2026-02-12

**Authors:** Eusebiu Catana, Francesca Merlo, Guido Perboli

**Affiliations:** 1 Ertico-ITS Europe, Brussels, Belgium; 2 Management and Production Engineering, Polytechnic of Turin, Turin, Piedmont, 10029, Italy

**Keywords:** Port Logistic, 5G technology, Digital transformation, Collaborative Policymaking

## Abstract

**Abstract:**

**Background:**

Seaports constitute strategic nodes in global supply chains and play a central role in the ongoing digital and green transition. Technologies such as 5G and artificial intelligence (AI) have the potential to enhance operational efficiency, safety, and environmental sustainability in logistics. However, their successful deployment depends not only on technological maturity but also on effective governance frameworks and coordinated stakeholder engagement. The 5G-LOGINNOV project addressed this intersection between innovation and governance by linking technical experimentation with policy learning to support the transition toward smart ports.

**Methods:**

The study employed an integrated methodological framework that combines the Collaborative Governance Regime (CGR) with the GUEST lean business methodology. This approach facilitates sustained multi-stakeholder collaboration and ensures that experimental outcomes are translated into validated business models and policy recommendations. Empirical evidence was collected from three Living Labs—Athens, Hamburg, and Koper—through workshops, interviews, and surveys involving private and public sector actors.

**Results:**

The integrated CGR–GUEST approach led to the co-creation of policy frameworks guiding 5G deployment in ports and hinterland networks. It identified major implementation barriers—technical, infrastructural, regulatory, and organizational—and produced actionable recommendations to address them. The framework also enhanced the comparability of pilot results and supported the translation of innovation outcomes into policy-relevant insights.

**Conclusions:**

The findings demonstrate that 5G deployment in logistics is not merely a technological endeavor but a governance challenge. By integrating structured governance mechanisms with lean innovation tools, the proposed framework bridges experimentation and policy, offering a replicable model for aligning disruptive technologies with broader sustainability and competitiveness objectives.

## Plain Language Summary

This study explores how new technologies such as 5G and artificial intelligence (AI) can make ports smarter, safer, and more sustainable. Ports are key hubs in global trade, and improving their efficiency is essential for both the economy and the environment. However, adopting advanced digital systems is not only a technical task—it also requires coordination between many public and private actors and clear policies to guide the process.

The research looked at how to bridge this gap between innovation and governance. It introduced a new approach that combines collaborative decision-making with a lean method for turning experimental results into practical business and policy tools. This approach was tested in three European ports—Athens, Hamburg, and Koper—where 5G and AI were deployed in real operating conditions.

Through workshops, interviews, and surveys, the study identified common challenges such as outdated infrastructure, regulatory uncertainty, and limited technical expertise. It then developed concrete recommendations to help ports, companies, and policymakers overcome these barriers.

The findings show that the success of digital transformation in ports depends as much on governance and cooperation as on technology itself. By aligning innovation with clear policy frameworks, ports can better use 5G and AI to improve logistics, reduce emissions, and support Europe’s transition toward smarter and greener transport systems.

## 1. Introduction

Seaports have a central position in the global economy and function as indispensable nodes in international supply chains. Maritime transport moves over 80% of the world’s traded goods by volume according to the most recent UNCTAD Review of Maritime Transport and associated seaborne trade statistics ( UNCTAD, 2025). Ports are important not only for cargo handling, but also as logistical hubs, industrial clusters, and gateways to hinterland networks. The pressure to improve and digitalize comes from growth in cargo volumes, increase in vessel sizes, and environmental regulations. Digital technologies—particularly Fifth Generation (5G) wireless communication and artificial intelligence (AI)—are emerging as critical enablers of efficiency, safety, and sustainability ( Lu & Xu, 2024; Zhou *et al.*, 2024). After 5G provides the high-capacity, ultra-low-latency connectivity required for real-time data exchange and mission-critical applications, AI can leverage this data to enable predictive analytics, automation, and decision support, making the two technologies complementary.

These developments align closely with the European Union’s digitalization agenda, the Green Deal, and the Industry 5.0 vision and their goals to enhance the resilience, competitiveness, and environmental performance of transport and logistics. The transition toward smart ports is not a simple technological upgrade but a fundamental change in how ports operate, manage resources, and integrate with global supply chains. This transformation is driven by a confluence of pressures: accommodating larger and more frequent vessels, managing increasingly complex cargo flows, reducing operational costs, minimizing environmental impact, and maintaining the highest safety standards ( Nguyen & Kim, 2024).

The paper is organized as follows: Section 2 explains our research objectives and the relevance of our study, Section 3 gives an overview of the existing literature, Section 4 describes the 3 living labs from the project 5G-LOGINNOV, Section 5 introduces our methodological framework combining the CGR and the GUEST, Section 6 details the results of our work and the proposed recommendations for policymakers, Section 7 summarizes and discusses the results and the implications for the future.

## 2. Research gaps and paper contribution

The format of the main body of the article is flexible: it should be concise, making it easy to read and review, and presented in a format that is appropriate for the type of study presented.

### An integrated methodological framework

To bridge the gap between technological potential and governance needs, this paper proposes a combined methodological approach. The Collaborative Governance Regime (CGR) framework offers a lens to promote trust, shared motivation, and iterative problem-solving among diverse stakeholders, while the GUEST methodology introduces a lean, standardized process for business modeling and innovation deployment, ensuring that co-created visions are transformed into replicable outputs. We believe these approaches can provide a structured yet flexible foundation for stakeholder collaboration, policy co-creation, and business case validation across different operational contexts.

### Research objectives

This study pursues two interrelated objectives. First, it aims to co-create policy frameworks and recommendations to guide 5G deployment in ports and hinterland networks. Second, it seeks to demonstrate a replicable governance model that aligns technological innovation with public policy objectives in complex, multi-stakeholder contexts. To do so, it leverages empirical findings from the 5G-LOGINNOV Living Labs, such as IoT and AI integration in Hamburg to improve environmental performance, and enhanced workplace safety in Koper, and the business models derived from these experiences.

### Contribution and relevance

By combining empirical evidence from the 5G-LOGINNOV Living Labs with a structured governance methodology, this research contributes practical policy guidance for European ports and policymakers, while also presenting a governance approach that can be adapted to other critical infrastructures facing similar challenges in adopting disruptive technologies. The recommendations developed in this study include technical feasibility and organizational and socio-economic considerations. In doing so, they respond to the urgent need for integrated approaches that connect innovation potential with implementation capacity. The final goal is to enable 5G to become as a driver of smarter, safer, and more sustainable port ecosystems.

## 3. Literature review

Literature on 5G adoption in smart ports demonstrates its potential to improve operational efficiency, enable real-time data processing, and strengthen safety measures. However, consistently achieving ultra-low latency and overcoming infrastructure limitations remain key barriers. Gaps in regulatory frameworks and limited investment incentives also delay large-scale adoption. Addressing these challenges is essential to accelerate smart port development and unlock 5G’s full potential in maritime logistics.

### The benefits of 5G in ports

Ports are a complex environment, with high levels of interference. 5G technology allows for the reliable transmission of vast amounts of data with wireless communication even in these difficult conditions. Its transformative role extends across several domains, like automation, where 5G can enable sophisticated control and remote operation of cranes, automated guided vehicles (AGVs), and other critical assets ( Torsoli *et al.*, 2023). The introduction of digital twins further strengthens operational capacity: high-bandwidth communication supports real-time digital replicas of port operations, enhancing optimization and predictive analytics ( Yigit *et al.*, 2023). Similarly, supply chain visibility benefits from continuous monitoring of containers, vessels, and equipment, which brings unprecedented levels of transparency across the entire port ecosystem ( Dolgui & Ivanov, 2022). Safety and reliability are also enhanced, since ultra-reliable low-latency communications allow precise operation of heavy machinery and autonomous vehicles in dynamic port settings ( Kokkoniemi-Tarkkanen *et al.*, 2019). 5G does not simply represent an incremental improvement over previous generations of wireless technology but is actually an enabling infrastructure that supports entirely new operational paradigms. Nevertheless, many studies privilege technological novelty over the development of viable business models, thus limiting understanding of economic feasibility and practical deployment challenges ( Potter *et al.*, 2025).

### Evidence from testbeds and pilots

Many research projects have deployed a wide range of real-life tests in ports and hinterlands to validate 5G applications. The 5G-Blueprint project and trials at the Port of Antwerp have demonstrated 5G’s capability to provide ultra-low latency and high reliability for teleoperation and vessel control ( Slamnik-Kriještorac *et al.*, 2023). Additional simulation and field tests have confirmed the potential of network slicing, edge computing, and private 5G networks specifically tailored to port environments ( Andriani *et al.*, 2023; Charpentier *et al.*, 2024). Trials in Bristol and Riga have explored more targeted use cases, including the installation of sensors in containers to track the location and condition of cargo ( Potter *et al.*, 2025) and the use of drones for safety and security operations ( Ferro *et al.*, 2020).

### From technology to governance

Existing research on 5G in port environments remains largely technology-focused, concentrating on applications such as AGVs, IoT sensor networks, and digital twin platforms, while devoting limited attention to governance frameworks, policy design, and stakeholder coordination. Yet, the success of disruptive innovations such as 5G depends as much on policy and regulations as on technical performance. Without clear models of coordination, the risk is to follow fragmented innovation pathways that fail to leverage economies of scale or interoperability benefits. Several studies underline the need for stronger governmental and regulatory support to incentivize 5G deployment in maritime and port sectors, highlighting its role in innovation facilitation and public–private collaboration ( Catana *et al.*, 2023; Potter *et al.*, 2025). Other contributions stress the importance of aligned policies for spectrum allocation and cross-border teleoperation ( Slamnik-Kriještorac *et al.*, 2023; Trichias *et al.*, 2021). However, these insights remain scattered, and detailed analyses of policy frameworks specific to ports are still scarce. Gaps persist in the evaluation of infrastructure investment incentives, and regulatory support for private 5G networks in port environments, limiting the availability of actionable guidance for both policymakers and practitioners.

In this paper we address the gap between existing testbeds and pilots and the transferability of results into useful guidance for policymakers with our integrated CGR-GUEST framework.

## 4. Living Labs as testbeds for innovation

The 5G-LOGINNOV project demonstrated the transformative potential of 5G technology in three diverse ports: Athens, Hamburg, and Koper. In Athens, a private 5G network combined with edge computing, AI-driven video analytics, and connected port assets allowed real-time tracking, predictive maintenance, and safety-critical operations. Hamburg applied 5G-enabled solutions to optimise hinterland connectivity, traffic management, and carbon reduction, using innovations such as automated truck platooning and floating truck emission data to support its smart mobility and decarbonisation goals. Koper pioneered the integration of 5G Standalone and Non-Standalone infrastructures with slicing, AI-assisted monitoring, and custom industrial IoT devices to deliver assured performance, enhance security, and automate logistics processes. Together, these Living Labs showcased how tailored 5G deployments can unlock efficiency, safety, and sustainability in modern port logistics ( Catana *et al.*, 2023).

### Athens living lab

The Athens Living Lab at Piraeus Container Terminal focused on using 5G technology to transform port operations through real-time asset tracking, predictive maintenance, and enhanced safety systems. By deploying a private 5G network integrated with edge and far-edge computing, AI-powered video analytics, and connected port assets such as yard trucks, cranes, and IoT devices, the lab demonstrated how ultra-low latency and high uplink capacity can enable mission-critical applications, improve operational efficiency, and strengthen security within the port environment.

### Hamburg living lab

The Hamburg Living Lab concentrated on applying 5G-enabled solutions to optimize port–hinterland connections, enhance traffic management, and reduce emissions. Through use cases such as floating truck emission data, automated truck platooning, and dynamic environmental traffic control, the lab showcased how low-latency, high-capacity networks can facilitate real-time data exchange among multiple stakeholders. These innovations not only improved operational efficiency but also delivered measurable carbon emission reductions, aligning with the city’s broader decarbonization and smart mobility strategies.

The combination of truck platoons with Time-to-Green information led to a 17% carbon emissions reduction ( 5G-LOGINNOV Consortium, 2024).

### Koper living lab

The Koper Living Lab at the Port of Koper explored advanced 5G Standalone and Non-Standalone network deployments to enable Industry 4.0 applications for port logistics, security, and automation. By integrating dedicated spectrum, slicing capabilities, AI-assisted video analytics, drone-based monitoring, and newly developed industrial 5G IoT devices, the lab validated how tailored private networks can ensure assured QoS, enhance asset visibility, and support mission-critical services. Thanks to these developments Koper can be viewed as a model for secure, high-performance smart port operations ( 5G-LOGINNOV Consortium, 2024).

## 5. Methodology

The methodological design of this research is grounded in the recognition that the deployment of disruptive technologies in complex, multi-stakeholder ecosystems requires both governance innovation and structured operational tools. To address this challenge, the study adopts an integrated framework that combines the Collaborative Governance Regime (CGR) with the GUEST methodology. The CGR framework provides a theoretically robust foundation for fostering sustained stakeholder interaction, mutual trust, and collective problem-solving, thereby aligning with established scholarship on collaborative policy-making in contexts of institutional and organizational interdependence. Complementing this governance perspective, the GUEST methodology introduces a standardized and iterative process for business modeling and innovation management, enabling comparability across cases and the systematic derivation of policy-relevant outputs. In the following, we give a brief description of them.

### Integrated CGR–GUEST framework

This study employs an integrated methodological approach combining the Collaborative Governance Regime (CGR) with the GUEST methodology to generate policy recommendations with the knowledge gathered during the 5G-LOGINNOV project. CGR provides the governance structure for sustained, multi-stakeholder engagement, while GUEST delivers the process management tools to transform shared visions into actionable, tested outputs.

### Collaborative Governance Regime (CGR)

CGR can be applied to promote iterative engagement among governments, private sector actors, and civil society organizations. It has been used for public decision-making in contexts where collaboration between various stakeholders is necessary to carry out public objectives that cannot be reached by any single actor alone ( Emerson *et al.*, 2012). CGR is characterized by an iterative approach that allows for continuous feedback and adaptation and can be applied to various policy domain with different levels of complexity. Unlike short-term participatory approaches, CGR is based on a sustained interaction between the participants over time, creating cooperation and trust and improving collective problem-solving. The participants involved in CGR activities are engaged in a collaboration dynamic, which is split in three interacting components:

• Principled Engagement: Stakeholders align their interests through continuous and structured dialogue, discussions, and interactive workshops. The GUEST supports this phase by providing easy tools that can be used by non-professional people with different backgrounds and by reducing the risks of misunderstandings with uniform data and value definitions.
• Shared Motivation: Trust and commitment are built and reinforced through regular meetings, various engagement strategies, and iterative feedback mechanisms. The credibility is reinforced by GUEST’s transparent process.
• Capacity for Joint Action: It is developed by leveraging institutional arrangements, leadership, knowledge, and resources with the aim of building the ability to work together and implement solutions effectively

These components are not viewed as a linear sequence of steps but work together in a cyclical and iterative way, creating a virtuous cycle of collaboration, in a continuous effort to create value together.

### The GUEST methodology

One of the objectives of this work is to present a user-friendly methodology, applicable throughout the decision-making process, to facilitate the adoption of 5G-enabled solutions ( Brotcorne *et al.*, 2019; Fadda *et al.*, 2018). GUEST is a Lean Business methodology, originally formulated by a pool of researchers associated with Politecnico di Torino ( Perboli, 2016; Perboli, 2017; Perboli *et al.*, 2018), is a framework frequently designed to support organizations in contexts of rapidly evolving and disruptive innovation. It supports and guides project development ensuring the integration of both the technological and the business dimensions. A core strength of this methodology lies in its emphasis on comparability and iterative learning across multiple pilots, in line with business model research that advocates cross-case analysis to identify enablers and barriers to value creation. This aspect is particularly critical in the early phases of innovation, where failures tend to outnumber successes, as it helps highlight the components that require close monitoring ( Cantamessa *et al.*, 2018). Originally built on the foundational contributions of Osterwalder and Pigneur ( Osterwalder & Pigneur, 2010) and the Lean Startup movement, GUEST extends these principles to products and services operating within Multi-Actor Complex Systems (MACS). It is anchored in recognized frameworks (e.g., Technology–Organization–Environment, socio-technical transitions), and supported by expert feedback and best practices in blockchain consortia, thereby strengthening its credibility ( CONCORDIA Consortium, 2020; INCIT-EV Consortium, 2021; NOUS Consortium, 2023; Perboli *et al.*, 2018; SINFONICA Consortium, 2023). The GUEST has already been successfully applied in several sectors, including automotive, information technology and telecommunications where it guided companies in defining clear value propositions, stakeholder roles, and implementations ( 5G-LOGINNOV Consortium, 2021; CONCORDIA Consortium, 2020; INCIT-EV Consortium, 2021; NOUS Consortium, 2023; SINFONICA Consortium, 2023). By documenting this proven framework in a replicable manner, we aim to further stimulate empirical testing across additional contexts, potentially uncovering how factors such as regulatory shifts, cultural readiness, or market structures affect 5G technology Challenges **as** technical solutions on operational efficiency, sustainability, and helps the emergence of new business models. Our approach combines action research principles with expert-driven validation. Our methodology is articulated in five steps: Go, Uniform, Evaluate, Solve, and Test. Each step allows the actors to monitor their projects and, at the same time, grants the standardization of documents and tools that should be used to evaluate ideas, outcomes, actions, and results. One of the main advantages of the GUEST is that it promotes inclusivity: innovation teams can benefit from participants with varying levels of seniority and expertise, provided they share a common language. The methodology does not require prior expert knowledge, allowing people from diverse backgrounds to contribute effectively and push innovation through collaboration.

### Application in 5G-LOGINNOV

The GUEST methodology was applied extensively in the 5G-LOGINNOV project to align technical innovation with business and policy development. Its structured process enabled the efficient involvement of diverse stakeholders, including start-ups, SMEs, established project partners, and public authorities, ensuring that the emerging solutions were both market-oriented and policy-relevant.

The GO phase of the GUEST framework guided us in identifying stakeholders and understanding their main needs.

Different typologies of stakeholders were identified: Port authorities and operators, Technology providers and MNOs, SMEs and start-ups (Open Call winners), Research institutes, Policy and standardization bodies (ALICE, CCAM, 5G-PPP).

They were selected and engaged according to the following criteria:

- Direct involvement in the Living Lab deployment
- Decision-making authority over infrastructure or regulation
- Ability to validate business and policy relevance

***Roadmap review and policy definition***

The process began with CGR-led technical workshops, where stakeholders jointly assessed existing logistics roadmaps (such as the ERTICO Logistics Roadmap 2025) and related frameworks. Representatives from port authorities, terminal operators, technology providers, SMEs, research institutions and policy networks were involved. These workshops followed a structured format merging roadmap presentation with facilitated discussions, to provide a shared understanding of ongoing trends and gaps in the logistics sector, based on emerging evidence from the Living Labs. After clustering and validating the results, we formulated two primary policy frameworks to structure the following steps of the work.

***Challenge categorization***

Leveraging GUEST’s standardization tools within CGR’s collaborative environment, stakeholders analyzed the experiences and feedback from the Living Labs, including workshop outputs, surveys, and use-case reports. To complement evidence from the Living Labs the innovation radar was used to assess the technological readiness, the business orientation and possible exploitation pathways of the different use-cases and the innovations in development.

This effort led to the identification of six key challenge categories, which acted as a reference taxonomy for organizing barriers and opportunities in 5G-enabled logistics innovation.

***Stakeholder engagement and business modelling***

The UNIFORM phase of the GUEST methodology played a central role in mapping stakeholder expectations against their actual needs during the development of new business models. This process initially led to the definition of a few high-level business models through a scenario analysis. Then they were refined and detailed by incorporating Living Lab evidence and stakeholders’ feedback ( Musso *et al.*, 2022). This activity engaged not only the five start-ups selected through the 5G-LOGINNOV Open Call for Innovative Start-Ups but also the established project partners, including 11 SMEs from the logistic and telecom sector. By involving both new entrants and experienced actors, the methodology ensured that innovative solutions were developed focusing on both viability and ecosystem integration.

***Recommendation development***

In the “Evaluate” and “Solve” phases of GUEST, Business Model Canvases were built for each Living Lab use case to capture value propositions, stakeholder roles, implementation barriers and constrains and expected impacts. These canvases were complemented by partner prioritization exercises, which helped to identify the most impactful actions. As a result, an initial portfolio of 64 recommendations was generated (32 for each policy framework), systematically clustered under the six previously defined challenge categories.

***Refinement and validation***

To strengthen the robustness and policy relevance of the results, recommendations were refined through an iterative process involving surveys, semi-structured interviews, and workshops with external stakeholders and partner organizations. This multi-layered validation ensured that the recommendations not only reflected the technical feasibility of 5G-enabled logistics solutions but also addressed the policy needs of the EU and local authorities.

***Outcome of the integrated approach***

By merging CGR’s governance strengths with GUEST’s structured execution, the methodology delivered three key outcomes:

• Stakeholder-owned, evidence-based policy recommendations, ensuring legitimacy and buy-in.
• Standardized outputs across diverse pilots, which enabled cross-case analysis and comparability of results.
• A replicable and scalable process that can be applied to future contexts of technology–policy integration, beyond the scope of 5G logistics.

***Final output***

**Figure 1.**
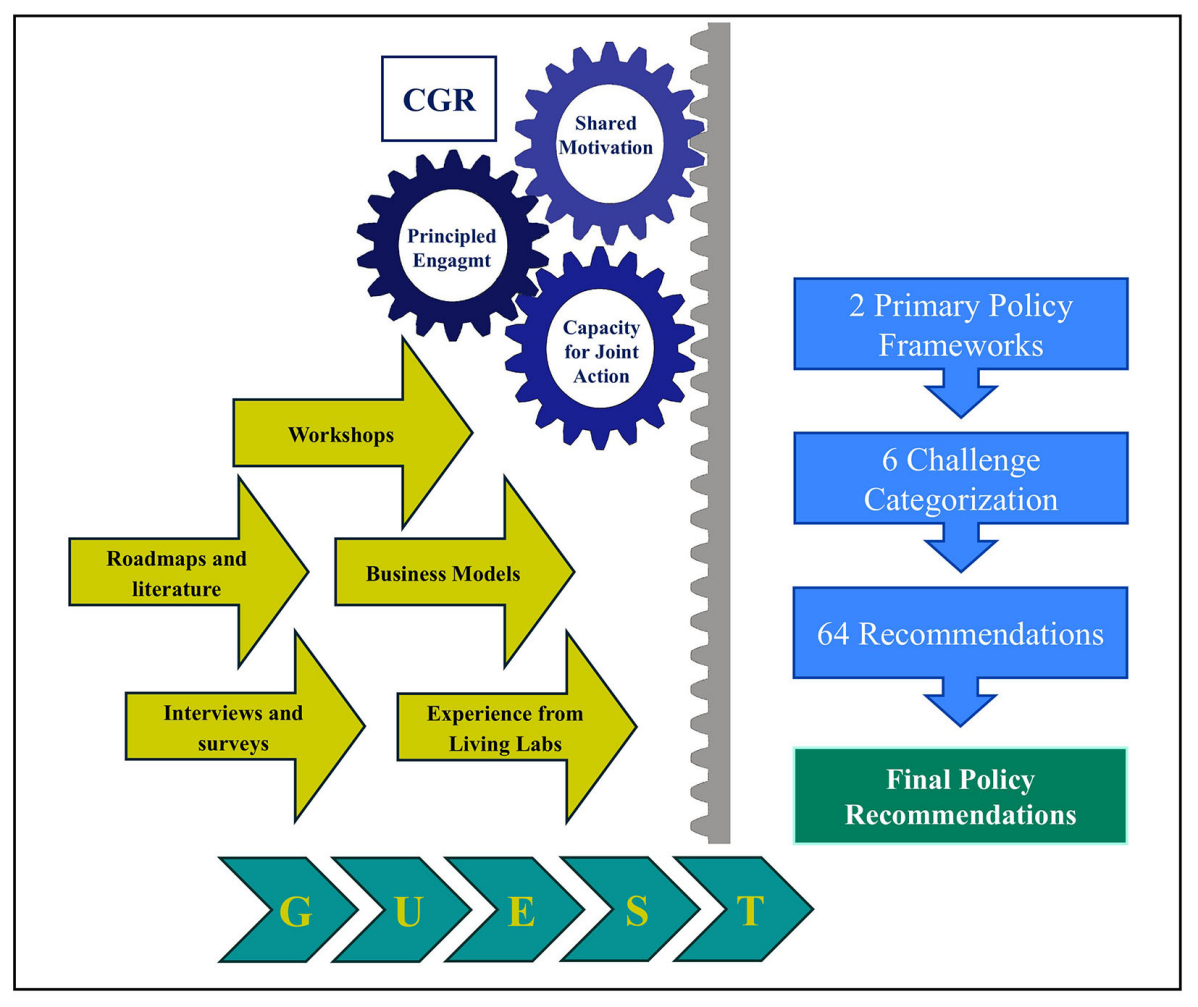
The methodological framework of our research.

The integration of results from various collaborative tools (e.g., surveys, interviews, workshops, and partnerships with external organizations) enabled the consolidation of a final set of recommendations. These were tailored for both public authorities and the European Commission, providing actionable guidance on how to support the adoption of 5G in logistics and how to overcome sectoral challenges through coherent policy measures. The entire process is summarized in Figure 1.

## 6. Results

### Key findings from technical workshops

**Figure 2.**
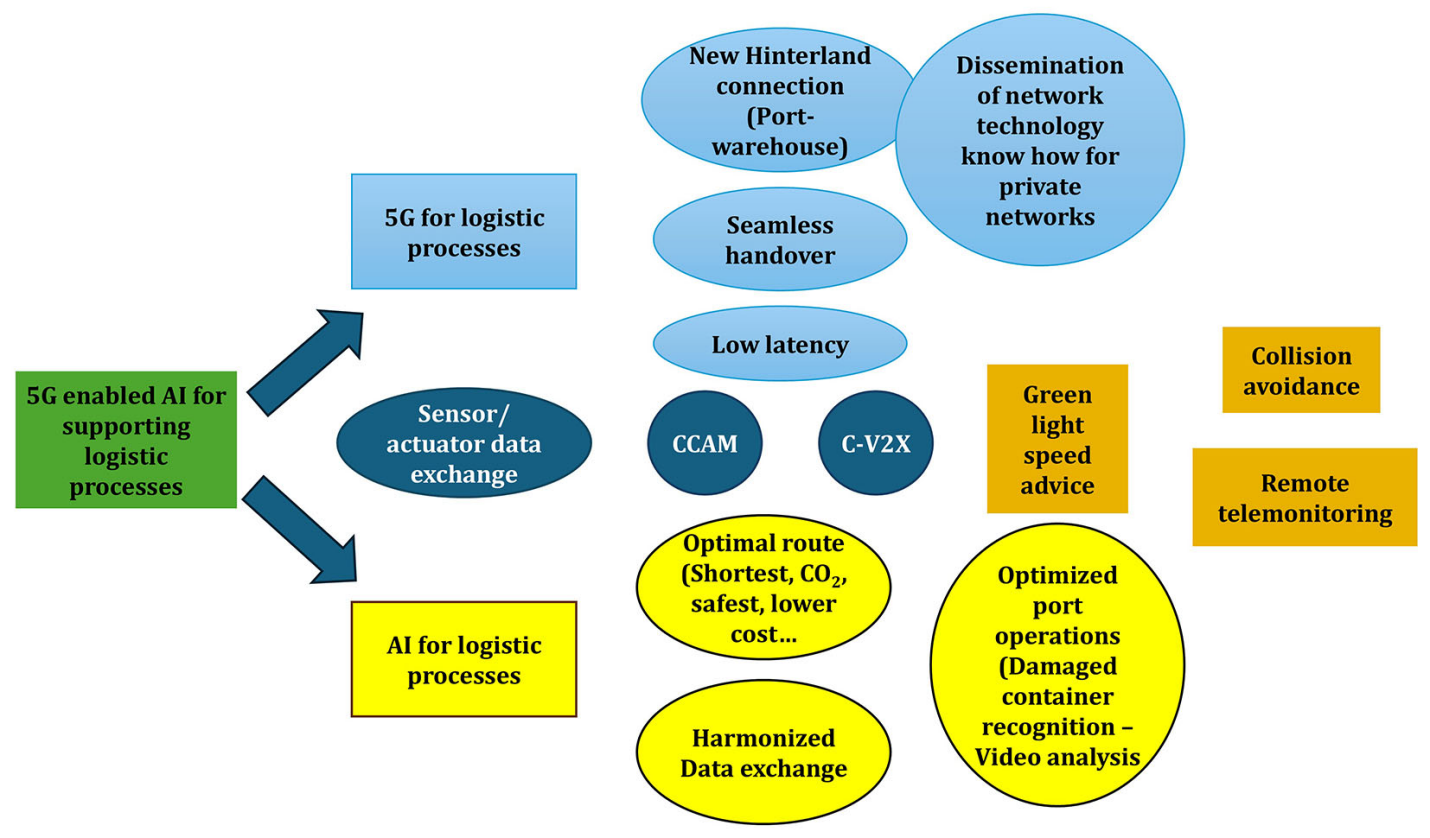
The Mind Map developed in the workshop.

The application of the integrated CGR–GUEST approach began with a collaborative assessment of existing logistics roadmaps. Among the documents reviewed, the ERTICO Logistics Roadmap 2025 emerged as the most relevant reference, as it highlights three strategic priorities for the sector: full digitalization, automation, and interoperability across transport and logistics systems. Building on this, the workshops examined how the outputs of the 5G-LOGINNOV project could align with and reinforce these priorities. Stakeholders identified 5G-enabled AI for logistics as the most significant innovation, complemented by advancements in green-light speed advice systems, collision-avoidance mechanisms, and remote telemonitoring solutions. To structure these findings, a high-level mind map ( Figure 2) was developed. This exercise distilled the results into two overarching policy frameworks that serve as the foundation for subsequent recommendations targeting public authorities and the European Commission:

• Ensure the priority roll out of 5G networks on EU hinterland network and port area
• Enhance research, development, and deployment of AI applications to support most optimal Logistic supply chain and port operation

These two policy directions directly enhance efficiency, safety, security, and sustainability in port operations and connected transport networks.

### Identification of deployment challenges

Through the integration of GUEST’s standardization tools within the CGR collaborative framework, a structured process was adopted to identify barriers to the implementation of the two policy directions. This process combined insights from stakeholder workshops with evidence gathered in the three European Living Labs. From the Innovation Radar 13 innovations were identified, with commercialization horizons between three and five years. This distribution confirms that 5G-enabled solutions are in a pre-scaling phase, and the importance of governance coordination and business model validations. The outcome was the formulation of six major categories of impediments, each reflecting practical issues and roadblocks encountered during project deployment. These categories, along with their brief descriptions, are summarized in Table 1.

**Table 1.** Summary of gaps identified.

Challenge	Description
Technical gaps	These encompassed technical problems such as inadequate electricity connections and compatibility issues with legacy infrastructure.
Infrastructure and Installation gaps	Problems arising from the lack of compatibility with legacy infrastructure were examined.
Regulatory gaps	Including instances where installation and operation didn’t align with existing laws, requiring approvals, or dealing with legislative gaps and unclear rules.
Administrative issues	Administrative concerns included the need for certificates, registrations, and compliance tests.
Training and Expertise	This addressed challenges related to the shortage of technical personnel to install equipment and difficulties in understanding its operation.
Business Models	This included observations of resistance from customers or entrepreneurs regarding investment in the project, possibly due to unclear business models.

### Development and prioritization of recommendations

The formulation of recommendations followed the Evaluate and Solve stages of the GUEST methodology. For each use case we developed a Business Model Canvas, integrating perspectives from both start-ups and established partners and ensuring a balanced representation of innovative and consolidated business approaches. This analysis produced an initial set of 64 recommendations, evenly distributed across the two policy frameworks and organized within the six impediment categories previously defined. The recommendations emerged from the examination of business models adopted in the Living Labs, with particular attention to the value propositions linked to the implementation of each use case. Partners were then invited to prioritize these propositions, a process that was further refined through Solution Canvas analysis and survey-based feedback, which allowed the most critical priorities to be clearly identified. In parallel, a comprehensive questionnaire was circulated to establish a shared vision on the main policy needs. It asked partners to reflect on the challenges encountered in deploying solutions in hinterland networks and port areas, on the degree of interest shown by 5G operators in supporting the project’s use cases, and on the potential measures to enhance capacity building for 5G equipment. The insights gained from this combined process of business model analysis, prioritization, and questionnaire validation were merged into a structured set of policy recommendations. Their final prioritization was integrated into the CGR governance cycle, ensuring both alignment and consensus across the consortium.

### External validation and refinement

The recommendations developed in the previous steps underwent an additional phase of validation and refinement through a series of exchanges with external stakeholders. In particular, two consultation workshops were organized in March and May 2023, complemented by two targeted interviews with representatives from ALICE, FENIX and CCAM. These activities provided new perspectives on challenges and possible solutions to 5G deployment, while also ensuring that the recommendations remained technically feasible and aligned with broader EU policy objectives. The first consultation workshop was focused on the 5G Infrastructure Public Private Partnership (5G-PPP). The session gathered representatives from the project’s Living Labs together with external delegates from the 5G-PPP community and focused on discussing the deployment gaps identified by 5G-LOGINNOV. The objective was to formulate shared recommendations that could be transferred to public authorities and the European Commission, strengthening the prospects for wider deployment and policy adoption. The second consultation workshop brought together a wider group of stakeholders, including representatives from CCAM, DTLF, FENIX and ALICE, alongside participants from the Living Labs. This workshop revolved around the two policy frameworks developed within the project and sought to identify concrete solutions to the gaps that might hinder the integration of 5G technologies in logistics. The discussions were aimed at supporting the implementation of 5G core technologies in European hinterland networks and ports, while also fostering the uptake of AI to improve efficiency in logistics operations. In addition to the workshops, individual interviews were conducted with representatives from ALICE, FENIX and CCAM. These interviews offered an opportunity to contrast the issues encountered during 5G-LOGINNOV deployments with experiences gained in other projects, and to explore possible strategies to address the identified roadblocks. The insights collected in these interviews were combined with the results of the workshops, reinforcing the robustness of the final set of policy recommendations.

### Final recommendations

The findings of the 5G-LOGINNOV project, consolidated through the GUEST–CGR methodology and validated with both internal and external stakeholders, led to the formulation of a set of final recommendations. These recommendations address the main challenges identified during the different use cases in the 3 Living Labs, and they provide guidance for public authorities, regulators, equipment manufacturers, and research communities. Their scope is twofold: on the one hand, they support the practical implementation of 5G and AI-based solutions in ports and hinterland areas; on the other, they contribute to broader EU objectives concerning digitalization, automation, interoperability, and sustainability in logistics.

***Reliable and Low-latency performance***

In hinterland regions served by commercially available 5G networks, the anticipated low-latency performance has not been consistently realized. Achieving reliable and sustained low-latency is essential for the effective operation of numerous critical applications. To address this challenge, it is imperative to foster close collaboration with Mobile Network Operators (MNOs) to mitigate license-related limitations, implement network slicing technologies, and streamline regulatory frameworks. Additionally, the integration of mobile edge computing infrastructure—such as localized data hubs at strategically positioned network nodes (e.g., Hamburg)—is recommended to minimize transmission delays and enhance system responsiveness.

***Compatibility of port infrastructure***

The integration of 5G hardware components within port facilities in some use cases has been delayed by structural and operational limitations. Cranes, identified as optimal sites for transponder installation, posed technical difficulties that necessitated reliance on legacy Wi-Fi systems, which offer limited coverage and performance. A transition towards 5G-enabled infrastructure is therefore essential. To facilitate this process, we propose the establishment of voluntary guidelines and standards for equipment upgrades, alongside incentives for port operators to invest in 5G- ready equipment. The promotion of innovation and competition in the private 5G market is further encouraged to ensure continuous technological progress.

***Standardize technical interfaces***

The absence of harmonized technical interfaces has resulted in fragmented solutions requiring ad hoc agreements with each supplier, thereby impeding efficient integration. To overcome these barriers, the creation of a Technical Standards Committee is recommended, tasked with defining open and interoperable standards for 5G deployment in ports and hinterland regions. Suggested measures include actions such as certification processes, early adoption incentives, international cooperation initiatives, and dedicated training programs and workshops. These policies are expected to simplify operations for service and equipment providers, reduce integration costs and foster market competition.

***Improve availability of AI tools***

Project partners encountered significant challenges in acquiring appropriate AI software solutions to support the planned applications. Despite the growing demand for AI technologies, there remains a notable scarcity of accessible, high-quality tools, which restricts both innovation and widespread adoption. Bridging this gap necessitates targeted investment in research and development, along with reforms in licensing and intellectual property frameworks to facilitate broader access. Emphasis should also be placed on establishing interoperability standards, ensuring that interfaces and documentation are intuitive and user-centric. Particularly crucial is the promotion of open-source initiatives, supported by both financial and institutional backing, as well as the creation of a centralized platform for the global exchange of AI toolkits and resources. Looking toward the future, AI generative models (LLMs) and Low-code/No-code Machine Learning Models (LMMs) are poised to play a transformative role. Their integration can democratize AI development, enabling a broader range of stakeholders—including non-experts—to create and deploy AI-driven applications. As these technologies evolve, it is vital to support their adoption by ensuring robust training datasets, developing ethical guidelines for generative AI, and fostering collaborative frameworks that allow users to harness these tools responsibly and effectively. Further investment in AI literacy and continuous feedback loops with AI communities will be necessary to ensure that these next-generation models align with the needs of industries and society at large.

***Deployment of dedicated cloud environments (Enhance Standardization)***

Security, privacy, and infrastructural constraints—such as restrictions on modifications to port cranes—posed significant challenges to the integration of AI tools within existing port ICT systems. To address these challenges, the adoption of dedicated cloud environments, such as Infrastructure-as-a-Service (IaaS) tailored specifically for port operations, is recommended. This approach would facilitate secure deployment while adhering to operational limitations. Furthermore, the development and promotion of standardized protocols and interfaces is essential for ensuring seamless interoperability between AI applications and port ICT infrastructure.

***Collaboration with equipment manufacturers***

Efficient logistics operations in ports often rely on the strategic positioning of devices—such as cameras on cranes—to support AI-driven services. However, issues of equipment ownership and the need to preserve manufacturer design specifications constrain deployment. Early and proactive engagement with equipment manufacturers is therefore required to co-develop solutions that are neutral, adaptable, and non-intrusive, thereby ensuring functional alignment with AI services while safeguarding the structural integrity of critical assets.

***Knowledge consolidation***

Finally, the absence of a centralized, sector-specific knowledge base for 5G applications in ports and hinterland areas represents a barrier to knowledge transfer. Establishing a comprehensive repository that consolidates research findings, case studies, and best practices relative to 5G in ports and hinterlands would serve as a valuable resource for stakeholders and foster informed decision-making to support a wider adoption of advanced digital technologies.

The recommendations are summarized in Table 2. Each entry highlights the key issue observed during project implementation and outlines the proposed actions to address it, with the overarching aim of fostering efficiency, reliability, and scalability in the deployment of 5G technologies in logistics.

**Table 2.** Final Recommendations for 5G Deployment in Ports and Hinterland Areas.

Recommendation	Key Issue Addressed	Proposed Actions
Ensure reliable low latency performance	Inconsistent latency in commercially available 5G networks	Collaborate with MNOs to resolve licensing barriers; introduce network slicing; streamline regulatory processes; deploy mobile edge hubs
Ensure compatibility of port infrastructure	Technical difficulties in installing 5G equipment on cranes; reliance on legacy Wi-Fi	Establish voluntary guidelines and standards; incentivize investment in 5G-ready equipment and infrastructure
Standardize technical interfaces	Lack of harmonized standards complicating integration	Create a Technical Standards Committee; define open standards; establish certification processes; incentivize early adoption; strengthen international cooperation; provide training
Improve availability of AI tools	Shortage of accessible, high quality AI software	Increase R&D investment; promote open-source initiatives; establish interoperability standards; reform IP and licensing; provide user-friendly interfaces; create centralized open-source platform
Deploy dedicated cloud environments	Security, privacy, and infrastructural restrictions hindering AI integration	Promote standardized protocols; deploy tailored IaaS solutions; ensure interoperability and secure integration of AI tools
Enhance collaboration with equipment manufacturers	Constraints on equipment placement and ownership; need for design neutrality	Engage manufacturers early; co-develop adaptable, nonintrusive equipment solutions; ensure alignment with AI service requirements
Create a centralized knowledge repository	Fragmented access to 5G-related resources for ports	Consolidate research papers, case studies, and best practices; establish a dedicated knowledge platform for stakeholders

### Added value of the CGR–GUEST integration

The integration of the CGR and GUEST methodologies provided significant added value to the project outcome: it ensured that all recommendations were developed with the engagement of all stakeholders and that they were grounded in validated business models. This ensured the legitimacy and the practical relevance of the recommendations. Finally, the establishment of continuous feedback loops between the governance-oriented CGR framework and the execution- oriented GUEST methodology accelerated the transition from the identification of challenges to the development and validation of consensus-based solutions.

## 7. Conclusions

This paper examined how the integration of the Collaborative Governance Regime (CGR) and the GUEST methodology can help regulators to guide the deployment of 5G technologies in European ports and hinterland networks. Combining structured governance with lean innovation tools we enabled the co-creation of policy frameworks, the prioritization of recommendations, and the validation of results across diverse Living Lab environments. Our findings show that 5G deployment in logistics is not only a technological challenge but also a governance issue. Challenges such as technical gaps, lack of standardization and fragmented knowledge must be addressed to support innovation and sustainability. Beyond the specific outcomes of the 5G-LOGINNOV project, this study contributes to the broader debate on digital transformation in critical infrastructures. It demonstrates that governance frameworks and methodological rigor are as important as technological advances in ensuring successful adoption.

### Limitations and boundary conditions of the CGR–GUEST framework

While the integration of the Collaborative Governance Regime (CGR) and the GUEST methodology proved effective in the context of the 5G-LOGINNOV Living Labs, its applicability is subject to some limitations. First, the framework requires sustained stakeholder engagement, repeated workshops and iterative validation cycles. This may not be feasible in contexts where timelines are constrained or competitive settings whit low trust between stakeholders.

Second, evidence was drawn primarily from EU-funded pilots, and the replicability of the approach in other regulatory, cultural, or market settings remains to be tested. A further limitation is the absence of feedback on the proposed policy frameworks. Since these recommendations are based on innovative, not-yet-implemented measures, their effectiveness can only be verified after these policies are enacted and evaluated in practice.

### Future research

Future research could therefore explore how governance–innovation frameworks can be adapted to non-European contexts or compare the evolution of barriers to 5G over time, which could affect the validity of policy recommendations. A potential direction for future research is to investigate how generative AI models—such as Large Language Models (LLMs) and Large Multimodal Models (LMMs)—can democratize AI development in ports, while ensuring ethical deployment, robust datasets, and alignment with industry needs to enable broader adoption.

## Ethics and consent

Ethic approval and consent were not required.

## Data Availability

The paper is based on evidence from the 5G-LOGINNOV project. Aggregated and anonymized open data are available on the 5G-LOGINNOV Zenodo community, including the following datasets:
- SeaFront – Synthetic dataset for visual container inspection. https://doi.org/10.5281/zenodo.10204550 ( Cortés *et al.*, 2024). This project contains the following underlying data:
•
SeaFront_v1_0_0.rar (synthetic image datasets for container classification and detection tasks). Data are available under the terms of the Creative Commons Attribution – NonCommercial – NoDerivatives 4.0 International (CC BY-NC-ND 4.0) license. - KPI set for evaluating the environmental impact of using 5G in data exchange for traffic management outside the port and the hinterland. https://doi.org/10.5281/zenodo.10204637 ( T-Systems *et al.*, 2024)
This project contains the following underlying data:
• Hamburg LL KPIs.csv (Key Performance Indicators measured during three trials at the Hamburg Living Lab of the 5G LOGINNOV project) • D2.2 – Data collection and evaluation procedures.pdf (Description of dataset collection and evaluation methodology) • D3.1 – Trial methodology, planning and coordination.pdf (Detailed trial methodology, planning and coordination document) Data are available under the terms of the Creative Commons Attribution 4.0 International (CC BY 4.0) license. (Zenodo) - KPIs to evaluate the benefits of 5G and AI-enabled services deployment on ports logistics operations. https://doi.org/10.5281/zenodo.10204619 ( Institute of Communication and Computer Systems *et al.*, 2024)
This project contains the following underlying data:
• D2.2 – Data collection and evaluation procedures.pdf (describes dataset structure and methodology) • D3.1 – Trial methodology, planning and coordination.pdf (details the trial setup and coordination) • dataset.zip (CSV files of KPI measurements including “A-KPI 11a – Inference time for both cloud and far-edge deployments (results per frame)” and “A-KPI 11b – Power consumption for both cloud and far-edge deployments (watts/second)”) Data are available under the terms of the Creative Commons Attribution 4.0 International (CC BY 4.0) license.
