# Supplementary material for: Governing digital transformation in ports: Policy learning from the 5G-LOGINNOV project
Source: Open Res Eur. 2026 Feb 12;5:362. Originally published 2025 Nov 26. [Version 3] doi: 10.12688/openreseurope.21641.3 (PMC12856245; doi:10.12688/openreseurope.21641.3)
Supplement: Peer review history

# Reviewer report: Reviewer response for version 2

**DOI:** 10.21956/openreseurope.24486.r68025

**Published:** 2026-01-29

**Authors:** Ioannis Filippopoulos

**Affiliations:** 1 University of Limassol, Limassol, Cyprus

The authors have revised the manuscript and have adequately addressed the main points raised in the previous round of review. The paper shows clear improvement in structure, clarity, and in the articulation of the CGR–GUEST framework and its relevance for policy analysis.

 While some aspects (particularly related to methodological operationalization and evidence traceability) could still be further strengthened, these remaining issues do not compromise the overall validity of the study. The manuscript remains timely and policy-relevant, and the integration of governance and innovation-management perspectives represents a substantive contribution to the literature on digital transformation in port and logistics systems.

 I therefore consider the revised version satisfactory and suitable for indexing.

Is the study design appropriate and does the work have academic merit?

Yes

Is the work clearly and accurately presented and does it cite the current literature?

Partly

If applicable, is the statistical analysis and its interpretation appropriate?

Not applicable

Are all the source data underlying the results available to ensure full reproducibility?

Partly

Are the conclusions drawn adequately supported by the results?

Partly

Are sufficient details of methods and analysis provided to allow replication by others?

Partly

Reviewer Expertise:

Maritime transport systems; smart ports and logistics; digitalisation of maritime and port operations; 5G and wireless communications in transport; AI, IoT, GIS and sensor-based monitoring; governance and policy aspects of digital transformation in maritime systems.

I confirm that I have read this submission and believe that I have an appropriate level of expertise to confirm that it is of an acceptable scientific standard.

# Reviewer report: Reviewer response for version 1

**DOI:** 10.21956/openreseurope.23410.r65353

**Published:** 2026-01-06

**Authors:** João Lemos Nabais

**Affiliations:** 1 Escola Superior de Ciências Empresariais, Instituto Politécnico de Setúbal, Setúbal, Portugal

The paper titled "Governing digital transformation in ports: Policy learning

 from the 5G-LOGINNOV project" addresses the digitalization process towards smart ports taking as reference the findings from the 5G-LOGINNOV project for the proposal of policy frameworks. The paper is interesting and relevant for the sector and can be improved throught the following remarks.

 Major comments:

 - page 6, in section "Collaborative Governance Regime (CGR)" the CGR is presented without any reference. Please review and include recent publications (in the last 5 years)

 - page 6, in section "Application in 5G-LOGINNOV" the application of the integrated CGR-GUEST Framework is explained but the alignment with the previous section is not clear. The CGR is presented decomposed into three components. The GUEST methodology is presented in five steps: Go, Uniform, Evaluate, Solve, and Test. The application is divided into: i) Roadmap review and policy definition ii) Challenge categorization iii) Stakeholder engagement and business modeling iv) Recommendation development and v) Refinement and validation. Figure 1 is not clear regarding the integration of CGR and GUEST, as it seems two independent processes. Please review and clarify.

 - Figure 2 "The Mind Map developed in the workshop." is not clear for presenting as stated in the paper "significant innovation, complemented by advancements in green-light speed advice systems, collision-avoidance mechanisms, and remote telemonitoring solutions." The figure includes terms that are not presented in the paper, namely CCAM and C-V2X. Please review the figure and improve the explanation for the findings in the text.

 - page 7, in section "Recommendation development" it is stated that "As

 a result, an initial portfolio of 64 recommendations was generated

 (32 for each policy framework), systematically clustered under the six previously defined challenge categories". 

 - page 9-10-11, in section "Final recommendations" the statement of the recommendations is not equal to the statement of recommendations in Table 2. Example: in the text-"Reliable and Low-latency performance"; in the Table- Ensure reliable low latency performance. Please review.

 - page 9, in section "Development and prioritization of recommendations" it is stated "Partners were then invited to prioritize these propositions, a process that

 was further refined through Solution Canvas analysis and survey-based feedback, which allowed the most critical priorities to be clearly identified." Please include information regarding the partners invited (for example: responsibilities, stakeholders, and number).

 - page 9, in section "Development and prioritization of recommendations" it is stated "In parallel, a comprehensive questionnaire was circulated to establish a shared vision on the main policy needs." Please include numerical data regarding the questionnaire (for example: number of submissions and a selection of meaningfull answers).

 - page 11, in Table 2 the last recommendation in accordance with the presentation in the paper should be revised to "Knowledge consolidation" and the statement "Create a centralized knowledge repository" is closer to a proposed action. Please review.

 Minor comments:

 - page 6, in section "Collaborative Governance Regime (CGR)" please verify the statement "[emerson2012integrative]."

 - page 6, section "The GUEST methodology" the statement "... or market structures affect 5G technology Challenges as..." please review the bold in "as"

 - page 8, Figure 8, in the block with the statement "Final Policy Recommendations" the number of Final Recommendations "7" helps the reader to relate the findings with Table 2. Please review.

 - page 9, in section "Final recommendations", it is stated "GUEST-CGR" please review for "CGR-GUEST" aligned with the order in which the integration between both is mentioned throughout the paper

 - page 10, "...environments(Enhance..." please review for "...environments (Enhance..."

Is the study design appropriate and does the work have academic merit?

Yes

Is the work clearly and accurately presented and does it cite the current literature?

Partly

If applicable, is the statistical analysis and its interpretation appropriate?

Not applicable

Are all the source data underlying the results available to ensure full reproducibility?

Partly

Are the conclusions drawn adequately supported by the results?

Yes

Are sufficient details of methods and analysis provided to allow replication by others?

Partly

Reviewer Expertise:

Digital Twin; Synchromodal Transport; Logistics; Supply Chain Management

I confirm that I have read this submission and believe that I have an appropriate level of expertise to confirm that it is of an acceptable scientific standard, however I have significant reservations, as outlined above.

# Reviewer report: Reviewer response for version 1

**DOI:** 10.21956/openreseurope.23410.r65894

**Published:** 2026-01-02

**Authors:** Saša Aksentijević

**Affiliations:** 1 University of Rijeka, Faculty of maritime studies, Rijeka, Croatia

**Summary:**

 The paper examines how the deployment of 5G and AI in seaports can be governed more effectively by integrating technological experimentation with collaborative policy learning. Drawing on evidence from the EU-funded 5G-LOGINNOV project and three Living Labs (Athens, Hamburg, and Koper), it proposes an integrated methodological framework that combines the Collaborative Governance Regime (CGR) with the GUEST lean business methodology. The core contribution lies in translating Living Lab experimentation into structured policy frameworks and recommendations for public authorities.

**Positive traits of the article:**

 The manuscript is generally well structured and clearly written, and it engages with recent and relevant literature on 5G, smart ports, and governance. 

 The overall study design is appropriate for an exploratory, governance-oriented investigation based on Living Labs and action research. The integration of the Collaborative Governance Regime and the GUEST methodology has academic relevance. 

 The paper refers to publicly available datasets and project deliverables, which supports transparency to some extent. 

 The conclusions are broadly consistent with the results and experiences described in the paper. 

**Negative traits of the article:**

 The study relies heavily on descriptive accounts of workshops, Living Labs, and methodological steps, but offers limited analytical depth in terms of systematically analyzing empirical data. While interviews, surveys, and workshops are mentioned, the paper does not clearly specify sample sizes, response rates, coding strategies, or analytical procedures.

 The paper frequently reads as a structured project report rather than an academic research article. Many sections focus on describing project activities, consortium processes, and outputs (e.g., number of recommendations produced) without sufficiently abstracting these into analytical insights or theoretical contributions.

 Although the CGR–GUEST integration is presented as effective and value-adding, the paper offers limited critical discussion of its weaknesses, trade-offs, or failure points. The conclusions briefly acknowledge contextual limitations, but there is little reflection on situations in which the framework might not work.

 In the results section, the discussion expands into speculative themes such as Industry 6.0, quantum computing, neuromorphic systems, and global AI ecosystems. These elements are only loosely connected to the empirical evidence presented and dilute the focus of the paper.

 The implications for non-European ports and governance systems remain underdeveloped. While replicability is claimed, the paper provides few concrete insights into how different regulatory regimes, market structures, or governance cultures affects the applicability of the framework.

 Several sections, particularly those describing the Living Labs and the methodology, repeat similar points using slightly different wording.

 Although datasets and KPIs are referenced, the paper does not meaningfully integrate quantitative performance results into the analysis. Readers are told that benefits such as emission reductions or efficiency gains were observed, but concrete figures or comparative indicators are largely absent from the narrative.

**Suggestion to authors:**

 To make the article scientifically sound, the authors must primarily strengthen methodological transparency by clearly specifying data collection and analysis procedures so that the study can be replicated, and by explicitly explaining how empirical evidence from the Living Labs is transformed into results and conclusions. The manuscript should more clearly distinguish research findings from project deliverables and sharpen its theoretical contribution by clarifying what is novel in the integration of CGR and GUEST beyond the specific project context. Empirical claims should be better supported through structured use of available data or comparative evidence, while limitations and potential biases inherent to stakeholder-driven, EU-funded pilots should be discussed more critically. Finally, speculative forward-looking elements not directly supported by the evidence should be reduced or more tightly grounded to maintain scientific focus.

Is the study design appropriate and does the work have academic merit?

Partly

Is the work clearly and accurately presented and does it cite the current literature?

Partly

If applicable, is the statistical analysis and its interpretation appropriate?

Not applicable

Are all the source data underlying the results available to ensure full reproducibility?

Partly

Are the conclusions drawn adequately supported by the results?

Partly

Are sufficient details of methods and analysis provided to allow replication by others?

No

Reviewer Expertise:

Logistics and management in seaports, intelligent transport systems, cybersecurity, resilience, disruptive digital technologies R&D

I confirm that I have read this submission and believe that I have an appropriate level of expertise to confirm that it is of an acceptable scientific standard, however I have significant reservations, as outlined above.

# Reviewer report: Reviewer response for version 1

**DOI:** 10.21956/openreseurope.23410.r65895

**Published:** 2026-01-02

**Authors:** Ioannis Filippopoulos

**Affiliations:** 1 University of Limassol, Limassol, Cyprus

**Summary**

 This paper examines how the integration of the Collaborative Governance Regime (CGR) and the GUEST methodology can support the governance of 5G deployment in European ports and hinterland networks. Drawing on evidence from the 5G-LOGINNOV Living Labs, the study develops policy frameworks, identifies key deployment challenges, and formulates a comprehensive set of policy recommendations. The topic is timely and policy-relevant, and the combination of governance and innovation-management perspectives represents a valuable contribution to the literature on digital transformation in port and logistics systems. While the overall approach is well-founded and appropriate for the stated objectives, several clarifications are required to strengthen methodological transparency, analytical coherence, and evidence traceability.

**Major comments**

**1. Alignment between contribution claims and preceding sections**

**In Section 2 (“Research gaps and paper contribution”)**, the *Contribution and relevance* paragraph clearly articulates the intended value of the study in terms of policy guidance, integrated recommendations, and broader applicability. The authors could more explicitly anchor these claims in the preceding literature review (Section 3) by clarifying which specific gaps in existing governance approaches the paper addresses, and how the proposed CGR–GUEST framework enables the translation of Living Lab evidence into transferable policy guidance. This would improve the traceability between identified gaps and the stated contributions.

**2. Methodological transparency and replicability**

**In Section 5 (“Methodology”)**, the integrated CGR–GUEST methodology is well motivated and conceptually appropriate for the study’s objectives. However, to support replication and strengthen scientific transparency, the authors should provide more explicit detail on how the framework was operationalised in practice. In particular, clarification is needed regarding stakeholder selection and roles, the number and type of engagement activities (e.g. workshops, surveys, interviews), the analytical procedures used to synthesise inputs, and the criteria by which challenge categories and recommendations were derived and prioritised.

**Minor comments**

**1. Introductory framing and references**

**In Section 1 (“Introduction”)**, several contextual statements would benefit from minor clarification and citation refinement. These include: (i) the statement on maritime transport carrying over 80% of global trade by volume (UNCTAD);(ii) the alignment with EU policy frameworks (EU digitalisation agenda, European Green Deal, Industry 5.0); and (iii) the claim that 5G and AI are critical enablers of efficiency, safety, and sustainability. The authors may consider citing the specific UNCTAD data release, adding one or two authoritative EU policy references (particularly for the EU digitalisation agenda), ensuring that all cited works are complete and accessible, and briefly clarifying the complementary roles of 5G (as connectivity infrastructure) and AI (as a data-driven optimisation capability) to improve technical precision.

**2. Literature synthesis and governance focus**

**In Section 3 (“Literature review”)**, the *“From technology to governance”* subsection appropriately highlights the dominance of technology-centric studies in the 5G port literature. The authors could more explicitly structure the discussion around key governance dimensions relevant to port environments (e.g. spectrum governance, procurement, OT ownership, liability, cybersecurity, data governance, interoperability, workforce skills) and clarify how these motivate the use of the CGR–GUEST framework.

**3. Living Labs – analysis, evidence, and transferability**

**In Section 4 (“Living Labs as testbeds for innovation”)**, while the Living Labs are clearly described and technically credible, the section would benefit from a more analytical synthesis highlighting common patterns, governance-related lessons, and differences across Athens, Hamburg, and Koper. In addition, several performance-related claims (e.g. emission reductions, assured QoS, safety-critical operations) would benefit from clarification regarding whether they are based on quantitative KPIs, qualitative assessments, or stakeholder perceptions. Finally, briefly distinguishing which insights are considered broadly transferable versus context-specific would strengthen the policy relevance of the Living Lab evidence.

**4. Evidence and performance claims**

**In Section 6 (“Results”)**, several performance-related claims would benefit from clarification regarding the nature of the supporting evidence. In particular:

 (i) in subsection *“Key findings from technical workshops”*, statements referring to efficiency, safety, and sustainability gains associated with the two policy frameworks (Figure 2); (ii) in subsection *“Final recommendations”*, claims concerning reliable low-latency performance, emission reductions, assured QoS, and security enhancements; and (iii) in Table 2, where issues such as inconsistent latency, emission impacts, and performance assurance are linked to proposed actions. For these cases, the authors could clarify whether the claims are supported by quantitative KPIs measured in the Living Labs, qualitative operational assessments, simulations, or stakeholder evaluations, and briefly indicate how such evidence informed the synthesis of results.

**5. Scope and future-oriented claims**

**In Section 6 (“Key findings from technical workshops”)**, the discussion following Figure 2 introduces concepts such as Industry 6.0, quantum computing, neuromorphic computing, and deep human–AI cognitive integration. While these perspectives are interesting, they extend beyond the empirical scope of the Living Labs and the results generated within the 5G-LOGINNOV project. The authors may consider clearly framing these elements as long-term outlooks or future research directions, or alternatively limiting the discussion to concepts directly supported by the project evidence, in order to maintain analytical focus and credibility.

**6. Conclusions – scope and status of recommendations**

**In Section 7 (“Conclusions”)**, the discussion of generative AI (LLMs) and Low-code/No-code ML models would benefit from being explicitly framed as a long-term research outlook, as these technologies were not investigated within the Living Labs. In addition, while the authors correctly note that the effectiveness of the recommendations can only be verified post-implementation, they could briefly clarify the current status of these recommendations (e.g. whether they have been communicated to or considered by public authorities, or remain advisory outputs awaiting adoption).

**Overall assessment**

 This is a well-motivated and policy-relevant study addressing an important gap in the governance of digital transformation in ports. With clearer methodological operationalisation, improved traceability between evidence and recommendations, and minor refinements to scope and framing, the paper would make a strong contribution to both academic and policy discussions. I support indexing after revision.

Is the study design appropriate and does the work have academic merit?

Yes

Is the work clearly and accurately presented and does it cite the current literature?

Partly

If applicable, is the statistical analysis and its interpretation appropriate?

Not applicable

Are all the source data underlying the results available to ensure full reproducibility?

Partly

Are the conclusions drawn adequately supported by the results?

Partly

Are sufficient details of methods and analysis provided to allow replication by others?

Partly

Reviewer Expertise:

Maritime transport systems; smart ports and logistics; digitalisation of maritime and port operations; 5G and wireless communications in transport; AI, IoT, GIS and sensor-based monitoring; governance and policy aspects of digital transformation in maritime systems.

I confirm that I have read this submission and believe that I have an appropriate level of expertise to confirm that it is of an acceptable scientific standard, however I have significant reservations, as outlined above.
